# Demystifying Computational Biology: A Scalable Drug Discovery Framework for Medical Curricula

**DOI:** 10.7759/cureus.112721

**Published:** 2026-07-15

**Authors:** Christopher O Campbell

**Affiliations:** 1 Research, Orlando College of Osteopathic Medicine, Winter Garden, USA; 2 Specialty Medicine, Orlando College of Osteopathic Medicine, Winter Garden, USA

**Keywords:** drug discovery, homology modeling, medical training, molecular docking, pathogen genomics, preclinical, project-based learning

## Abstract

As precision medicine becomes an integral part of clinical practice, medical curricula would benefit from the inclusion of accessible training in bioinformatics and computational biology. This project provides a scalable, stepwise pedagogical framework for introducing medical students to the drug discovery pipeline using high-impact, small-scale computational projects. Each research project involves using online genomic databases to identify and characterize genes of importance to pathogenesis using *Plasmodium falciparum* as a model organism. The methodology changes from sequence to structure and teaches students to use bioinformatics tools to characterize genes and then applies advanced protein modeling techniques such as homology modeling and structural assessment. Students use platforms such as AlphaFold (developed through a collaboration of Google DeepMind, London, UK, and the European Molecular Biology Laboratory, Wellcome Genome Campus, Hinxton, Cambridgeshire, UK), SWISS-MODEL (Computational Structural Biology Group, Swiss Institute of Bioinformatics, Biozentrum, University of Basel, Basel, Switzerland), and SwissDock (developed through a collaboration between the Molecular Modeling Group of the University of Lausanne and the SIB Swiss Institute of Bioinformatics in Lausanne, Switzerland) to evaluate protein structures and model binding interactions with small molecules obtained from the PubChem and ChEMBL-NTD databases. The inclusion of these virtual screening protocols in these projects serves to demystify the complexities of computational biology, giving medical students concrete insights into the identification and translation of molecular inhibitors into potential therapeutic leads. The modular nature of this approach shows that complex concepts in drug discovery can be made accessible and relevant for future clinicians, thus cultivating the data literacy necessary to navigate the future of genomic medicine and infectious disease management.

## Introduction

The rapid integration of genomic medicine, targeted therapeutics, and precision medicine into clinical workflows demands a paradigm shift in undergraduate medical education. Modern physicians are not merely consumers of clinical guidelines; they must be data-literate clinicians capable of interpreting molecular data and understanding the preclinical foundations of the pharmacotherapy they prescribe [1-4]. However, traditional medical curricula often relegate bioinformatics and computational biology to isolated, lecture-based blocks, separating abstract molecular theory from practical clinical application.

In order to understand the importance of this paradigm shift, it is necessary to define the key digital technologies used in this review: bioinformatics, computational biology, and homology modeling. Bioinformatics is the use of computer science and statistics to store, organize, and interpret large libraries of biological data, such as genomic sequences. Computational biology goes further by constructing mathematical models and analytical simulations to study the behavior and function of complex theoretical and experimental biological systems. Homology modeling is an important structural bridge in the absence of a physical, three-dimensional structure of a disease-causing protein target; it predicts its three-dimensional molecular shape based on its genetic similarity to known, established protein templates.

In modern drug development, these combined tools completely revolutionize the traditional laboratory pipeline. Instead of physically testing millions of physical chemical compounds against a pathogen, researchers use these computational tools to rapidly screen digital libraries of millions of molecules. By virtually simulating how these molecules fit and bind into a pathogen's structural target pocket, this technology significantly accelerates the identification of viable therapeutic leads, dramatically lowers initial research and development costs, and transforms the timeline for delivering novel, life-saving treatments from decades down to months.

This review introduces a scalable, project-based learning (PBL) framework designed to fit within preclinical medical curricula or asynchronous research electives. This modular framework demystifies the drug discovery pipeline by transitioning from sequence to screening. Through this framework, students actively engage with the tools that define modern biomedical innovation.

Using *Plasmodium falciparum*, the primary causative agent of severe malaria, as a pedagogical model, this review will provide an example of the process students will use to explore pathogen genomics while mastering the fundamentals of rational drug design. Malaria remains a critical global health priority, and studying its evolving drug-resistance pathways offers an ideal clinical hook for future physicians learning to navigate infectious disease management and global health epidemiology. The documented expansion of artemisinin partial resistance across East Africa underscores an immediate clinical crisis, reinforcing the need for continuous genomic surveillance and novel drug discovery paradigms [5]. The project structure can be broken down into a three-phase workflow, taking the students on a path starting with target identification and eventually concluding with screening through molecular docking.

## Technical report

Phase 1: target identification (pathogen genomics)

The workflow begins at the foundational level of genomic translation. In this phase, students act as genomic investigators, navigating public repositories to identify viable therapeutic targets within the *P. falciparum* genome. The key pedagogical objectives can be broken down into three categories: understanding structural vs. functional genomics in pathogens, navigating online primary genomic databases, and assessing target druggability based on evolutionary conservation and host-pathogen homology.

Students utilize PlasmoDB (the *Plasmodium* genomics resource) alongside the National Center for Biotechnology Information (NCBI) BLAST tool [6,7]. The primary educational utility framework of each database is outlined in Table 1. The core task is to identify specific genes essential to the parasite’s lifecycle or pathogenesis, such as those involved in apicoplast metabolism, merozoite invasion, or hemoglobin degradation, while ensuring minimal homology to human protein counterparts to reduce potential off-target drug toxicity [8].

**Table 1:** Summary of open-access bioinformatics databases and molecular modeling tools integrated into the computational biology and drug discovery framework NCBI, National Center for Biotechnology Information; NMR, nuclear magnetic resonance

Database/Tool	Primary Educational Utility in Framework
PlasmoDB (The Eukaryotic Pathogen, Vector, and Host Informatics Resources (VEuPathDB), University of Pennsylvania, Philadelphia, PA, and University of Georgia, Athens, GA)	A specialized functional genomic database that integrates genomic, transcriptomic, and proteomic data specifically for *Plasmodium* species to facilitate malaria research.
NCBI BLASTp (National Center for Biotechnology Information, Bethesda, MA)	A sequence alignment tool that compares an amino acid query sequence against a protein database to identify similar sequences and evaluate evolutionary relationships or potential cross-reactivity.
SWISS-MODEL	An automated web server dedicated to homology modeling of protein 3D structures, allowing users to generate structural models based on known evolutionary templates.
Protein Data Bank	The primary global repository for three-dimensional structural data of large biological molecules, such as proteins and nucleic acids, determined via experimental methods like X-ray crystallography and NMR spectroscopy.
SwissDock	An online web service designed to simulate and predict molecular docking interactions between small-molecule ligands and protein targets to gauge binding affinity.
AlphaFold	An advanced AI system developed by Google DeepMind that predicts highly accurate three-dimensional structures of proteins directly from their genetic sequences.
Simple Modular Architectural Research Tool (SMART) (European Molecular Biology Laboratory, Heidelberg, Germany)	A web-based tool used for the identification and annotation of protein domains and the analysis of protein domain architectures.
Conserved Domain Database	An NCBI resource that provides annotation of functional units within protein sequences by identifying conserved structural domains and specialized motifs.
Kyoto Encyclopedia of Genes and Genomes (KEGG)	A comprehensive database resource that integrates genomic, chemical, and systemic functional information to map molecular interaction and reaction networks.
PubChem	An open chemistry database maintained by the NCBI that provides comprehensive information on the chemical structures, properties, and biological activities of millions of small molecules.
ChEMBL-NTD	A specialized subset of the ChEMBL bioactivity database dedicated to sharing chemical structures and screening data targeted specifically at neglected tropical diseases like malaria.
PyMOL (Schrödinger, Inc., New York, NY)	PyMOL is a premier molecular visualization software system used to view, manipulate, and render 3D images of biological macromolecules like proteins, DNA, and RNA, as well as small chemical compounds.

Phase 2: characterization (homology modeling and structural assessment)

Once a genomic target is selected, students transition from primary sequence strings to three-dimensional structural biology. Understanding that a drug interacts with a physical structural pocket, not a linear text sequence, is a critical milestone in medical data literacy. When experimental structures (via X-ray crystallography or nuclear magnetic resonance (NMR) spectroscopy) are unavailable in the Protein Data Bank (PDB), students leverage advanced predictive computational tools, such as AlphaFold, where students retrieve high-confidence, AI-predicted structural models to analyze structural domains and folding architectures [9]. Students also utilize tools such as SWISS-MODEL to study specific mutant strains or uncharacterized variants through traditional homology modeling. They select structurally resolved templates, align sequences, and generate 3D coordinate models [10,11].

To ensure the target model is viable for downstream virtual screening, students conduct structural validation. Utilizing Ramachandran plot analysis (via tools like PROCHECK or MolProbity (Richardson Lab, Duke University, Durham, NC)), students evaluate the stereochemical quality of the protein backbone. They learn to identify favored, allowed, and disallowed phi/psi (ɸ/Ѱ) dihedral angles, distinguishing between structurally sound models and those with steric clashes that could compromise docking accuracy [12].

Phase 3: screening (molecular docking and virtual screening)

The final phase of the framework simulates clinical pharmacology and preclinical trial design through computational molecular docking. Students explore the chemical space by curating potential inhibitory compounds from specialized small-molecule repositories. One such database is PubChem, which is used for identifying generalized chemical structures and established inhibitors [13]. They also use ChEMBL-NTD (Neglected Tropical Diseases) to browse a targeted repository containing chemical series and screening data explicitly focused on malaria pathogens [14,15].

**Figure 1:**
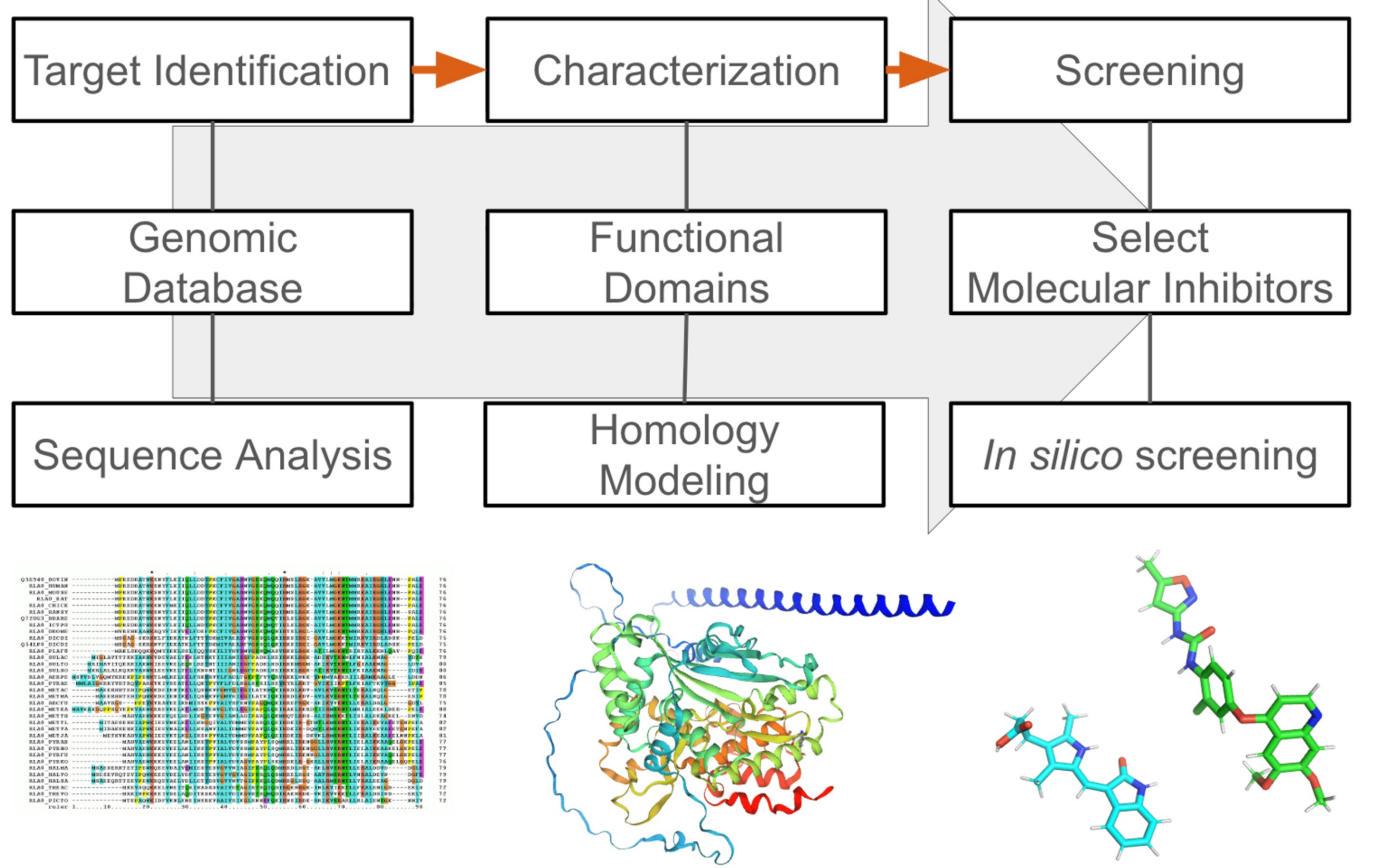
Molecular screening and docking workflow The molecular screening and target identification workflow. This example is using a protein kinase from *P. falciparum* obtained from PlasmoDB; however, the methodology can be applied to any pathogen using any genomic database. The process starts with target identification from the database. In this step, the sequence data from the database is reviewed to develop a background understanding of the gene, as well as any orthologs present in the genome. Characterization follows a process of deeper understanding of the functional domains or any specific signature motifs. From this data, similar structures are identified, and homology modeling is done. Screening is the process by which online databases of drug compounds are screened against the target protein. This figure was assembled using Google Slides (Google LLC, Mountain View, CA); the multiple alignment component was made with CLUSTALw (Kyoto University Bioinformatics Center, Kyoto, Japan); and the molecular image components were made using PyMOL (Schrödinger, Inc., New York, NY).

Using a structured workflow (Figure 1), students perform docking simulations using tools such as SwissDock or AutoDock using virtual screening protocols [16-18]. They define the structural grid coordinates around the protein’s active site or predicted binding pocket and initiate the docking simulation.

**Figure 2:**
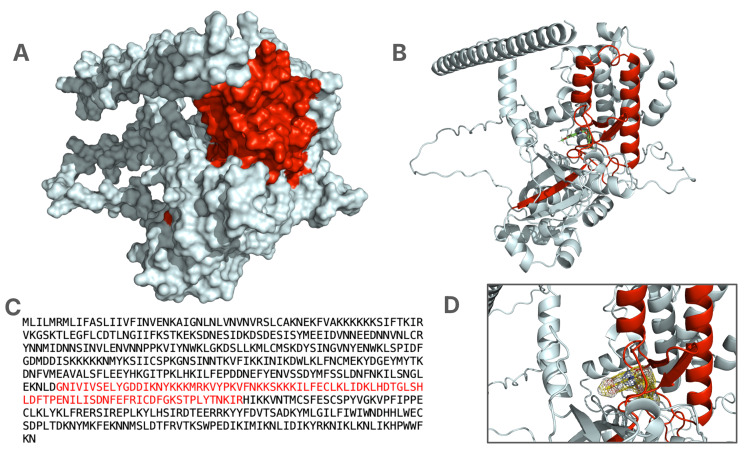
Evaluation of the functional domain and binding pocket This figure provides an overview of the evaluation of the functional domain of a *P. falciparum* kinase. In this example, a domain analysis highlights the specific region on the homology model where the proposed binding pocket can be found. Surface representation (A) and ribbon diagram (B) show the location of the functional region mapped on the homology model. The unique region of the sequence that aligns with known kinases is colored in red on the primary sequence (C). A closer view of the binding pocket gives a theoretical orientation of the region where residues interact with a potential ligand (D). This figure was assembled using Google Slides (Google LLC, Mountain View, CA); the protein sequence component was obtained from the PlasmoDB (The Eukaryotic Pathogen, Vector, and Host Informatics Resources (VEuPathDB), University of Pennsylvania, Philadelphia, PA, and University of Georgia, Athens, GA); and the molecular images were made using PyMOL (Schrödinger, Inc., New York, NY).

Students analyze the thermodynamic outputs, focusing on the Gibbs free energy (ΔG) of the binding conformation [19,20]. They learn to interpret highly negative binding affinity scores (kcal/mol) as markers of thermodynamically favorable interactions. Finally, using molecular visualization software (such as PyMOL), students visually inspect the binding posture, mapping specific hydrogen bonds, hydrophobic interactions, and electrostatic fits between the small molecule and the target amino acid residues. Figure 2 shows an example of the homology model developed and the mapping of the proposed binding site.

## Discussion

This educational framework demonstrates that sophisticated, translationally relevant computational biology can be successfully integrated into preclinical medical training without requiring extensive coding prerequisites. By making bioinformatics tools accessible in this modular format, we effectively synthesize three foundational pillars of traditional medical education: microbiology/infectious disease, biochemistry/pharmacology, and evidence-based medicine. In this structural approach, students shift from passive consumers of pharmacological data to active investigators. By analyzing pathogen survival and life-cycle vulnerabilities at the genomic scale, they gain a firsthand understanding of molecular resistance and structural biology. Rather than viewing pathogen mechanics as static illustrations, students utilize genomic data to actively map out life-cycle vulnerabilities and virulence factors. This hands-on exploration provides an immediate, structural context for understanding how specific genetic mutations manifest as clinical drug resistance.

Furthermore, the use of interactive docking simulations and structural modeling allows students to visually demonstrate drug-receptor interactions, competitive inhibition, and structural pharmacophores. By transitioning from two-dimensional chemical structures to dynamic 3D molecular docking simulations, this transforms how students conceptualize pharmacodynamics. Additionally, virtual ligand-receptor interactions, competitive inhibition, and the physical alignment of structural pharmacophores bridge the gap between abstract biochemical pathways and the tangible mechanism of action of a drug, shifting the learning paradigm away from the rote memorization of drug classes.

By interrogating primary genomic databases and evaluating predictive algorithmic outputs, students practice the core tenets of evidence-based medicine at a foundational level. They learn to critically appraise computational models, assess data limitations, and interpret statistical probabilities-skills that are directly translatable to navigating the rapidly evolving landscape of clinical literature and targeted therapies.

Ultimately, this framework does not aim to turn medical students into bioinformaticians, but rather to cultivate the critical evaluation skills required to navigate preclinical literature and future targeted therapies. As precision medicine and genomic surveillance become increasingly central to clinical practice, integrating scalable computational tools early in medical education ensures that the next generation of physicians is equipped to interpret, critique, and apply data-driven discoveries at the bedside.

## Conclusions

This learning framework relies on cloud-accessible, open-access tools with almost zero infrastructure costs. It is easily scalable from a small-group research track to a school-wide, asynchronous hybrid block that caters to diverse learning styles via interactive, self-paced computational execution.

Ultimately, by shifting from passive consumers of pharmacological data to active investigators of molecular inhibitors, future clinicians acquire the fundamental data literacy required to lead in the era of genomic medicine, preparing them for an evolving landscape of clinical practice and infectious disease management.
